# High Distribution of CD40 and TRAF2 in Th40 T Cell Rafts Leads to Preferential Survival of this Auto-Aggressive Population in Autoimmunity

**DOI:** 10.1371/journal.pone.0002076

**Published:** 2008-04-30

**Authors:** Gisela M. Vaitaitis, David H. Wagner

**Affiliations:** The Department of Medicine and Webb-Waring Institute, University of Colorado Denver, Denver, Colorado, United States of America; University Paris Sud, France

## Abstract

**Background:**

CD40–CD154 interactions have proven critical in autoimmunity, with the identification of CD4^lo^CD40^+^ T cells (Th40 cells) as harboring an autoaggressive T cell population shedding new insights into those disease processes. Th40 cells are present at contained levels in non-autoimmune individuals but are significantly expanded in autoimmunity. Th40 cells are necessary and sufficient in transferring type 1 diabetes in mouse models. However, little is known about CD40 signaling in T cells and whether there are differences in that signaling and subsequent outcome depending on disease conditions. When CD40 is engaged, CD40 and TNF-receptor associated factors, TRAFs, become associated with lipid raft microdomains. Dysregulation of T cell homeostasis is emerging as a major contributor to autoimmune disease and thwarted apoptosis is key in breaking homeostasis.

**Methodology/Principal Findings:**

Cells were sorted into CD4^hi^ and CD4^lo^ (Th40 cells) then treated and assayed either as whole or fractionated cell lysates. Protein expression was assayed by western blot and Nf-κB DNA-binding activity by electrophoretic mobility shifts. We demonstrate here that autoimmune NOD Th40 cells have drastically exaggerated expression of CD40 on a per-cell-basis compared to non-autoimmune BALB/c. Immediately *ex-vivo*, untreated Th40 cells from NOD mice have high levels of CD40 and TRAF2 associated with the raft microdomain while Th40 cells from NOR and BALB/c mice do not. CD40 engagement of Th40 cells induces Nf-κB DNA-binding activity and anti-apoptotic Bcl-X_L_ expression in all three mouse strains. However, only in NOD Th40 cells is anti-apoptotic cFLIP_p43_ induced which leads to preferential survival and proliferation. Importantly, CD40 engagement rescues NOD Th40 cells from Fas-induced death.

**Conclusions/Significance:**

CD40 may act as a switch between life and death promoting signals and NOD Th40 cells are poised for survival via this switch. This may explain how they expand in autoimmunity to thwart T cell homeostasis.

## Introduction

The role of CD40–CD154 interactions in autoimmune diseases [1], [2] including type I diabetes (T1D) [3]–[5], collagen induced arthritis [6], [7], systemic lupus erythamotosis [8], [9], and autoimmune thyroiditis [10], has been well documented. CD40 (tnfrsf5) belongs to the TNF-receptor family and when CD40 is engaged it becomes associated with lipid rafts to interact with the adaptor molecules, TNF receptor associated factors (TRAFs), for downstream signaling [11]. CD40 expression has traditionally been associated with B cells, dendritic cells and macrophages (antigen presenting cells, APC) [12] but has proven to be more ubiquitous and now has been described on T cells [6], [12]–[16]. In autoimmunity there is an expansion of a CD40-expressing T cell subset that also is characterized by its low surface-expression of CD4 [4], [15]–[17].

While the consequences of CD40 signals in APC have been extensively studied [12], [18]–[20] little is known about the outcome of CD40 signals in T cells. Specifically, do differences exist in the signaling outcome between autoimmune and non-autoimmune conditions? We previously demonstrated that CD40 engagement of a CD4^+^CD40^+^ T cell clone, BDC2.5, induced rapid activation of Nf-κB DNA binding activity [16] as well as induction of RAG1 and RAG2 expression, the proteins necessary for TCR expression [3]. Recently it was shown that engagement of CD40 on T cells can serve a costimulatory function [6]. The CD4^lo^CD40^+^ T cells, which we termed Th40 cells, are detected in diabetic and prediabetic NOD mice as well as in non-autoimmune controls [4], [15]. Interestingly, the Th40 cell subset is greatly expanded under autoimmune conditions and is necessary and sufficient in diabetes transfers from the non-obese diabetic (NOD) mouse to NOD.*scid* animals [4], [15], [16]. The expansion seen in autoimmune prone animals can be prevented by blocking CD40–CD154 interactions [4]. While the identical subset is present in non-autoimmune subjects, it remains contained at lower percentages of total lymphocytes [4], [15], [16].

Although the cause for peripheral T cell autoaggression in autoimmune disease [16], [21]–[25] is not fully understood, dysregulation of T cell control is emerging as a major contributor [4], [26], [27]. Specifically, loss of homeostasis between regulatory T cells (T_reg_) and effector T cells [26], [27], including potentially autoaggressive Th40 cells [4], is taking center stage. Homeostatic regulation of T cells is dependent on apoptosis [28], [29] and thwarted apoptosis of peripheral effector T cells is a key element in autoimmunity [28]. For example, mice deficient in Fas - FasL develop autoimmunity [30]. Events, such as engagement of death receptors ( e.g. Fas, TRAIL, TNF-R; i.e. extrinsic pathway) or loss of mitochondrial potential followed by release of cytochrome c (i.e. intrinsic pathway), can lead to apoptosis. The Bcl protein family (including anti-apoptotic Bcl-2 and Bcl-X_L_ and pro-apoptotic Bax and Bak) is involved in pro- and anti-apoptotic events in the intrinsic pathway while cFLIP, a homolog of caspase-8 and FADD that can act as a caspase-8 decoy, is involved in the extrinsic pathway [28], [29], [31], [32]. cFLIP can be both pro- and anti-apoptotic [33] but it can be cleaved to generate anti-apoptotic cFLIP_p43_ that interacts with TRAF2 and induces Nf-κB activation [34]. The two apoptotic pathways also converge as it was shown that Bcl-X_L_ expression affects the extrinsic pathway partly by inducing cFLIP expression [35].

Because we demonstrated a causal relationship of CD40–CD154 interactions leading to expansion of potentially autoaggressive Th40 cells *in vivo* [4], we investigated whether CD40-signals directly to the T cell can be responsible for such an expansion. Therefore we compared CD40 expression and signaling outcomes between autoimmune and non-autoimmune conditions as well as between Th40 cells and CD4^hi^ T cells within those conditions.

Here we show that Th40 cells from NOD, NOR and BALB/c mice have differences in CD40 protein expression levels as well as differences in CD40 and TRAF2 association with the raft microdomain. Immediately *ex-vivo* NOD Th40 cells are poised for CD40 signaling by having high amounts of CD40 and TRAF2 associated with the raft microdomain and induce high levels of Nf-κB activation as well as anti-apoptotic cFLIP_p43_ and Bcl-X_L_ protein expression in response to CD40 engagement. CD40 engagement of NOD Th40 cells increases survival and induces proliferation. CD40 engagement also rescues those cells from Fas-induced death. CD40-induced cFLIP_p43_ expression in NOD Th40 cells persists when Fas is simultaneously engaged. The same subsets from non-autoimmune NOR and BALB/c mice exhibit a difference in total levels of CD40 compared to each other but they both induce Bcl-X_L_ expression. However, neither NOR nor BALB/c T cells induce cFLIP_p43_ expression or proliferate in response to CD40 engagement. Therefore it is possible that CD40, via cFLIP_p43_, acts as a switch between Fas-induced cell death and survival/proliferation of these cells in autoimmunity. The results, together with our previous report that *in vivo* blockade of CD40–CD154 interaction in NOD prevents the expansion of potentially autoaggressive Th40 cells [4], suggest that a fundamental difference in CD40 expression and signaling leads to dysregulation of peripheral T cell control and may be a major cause for disease progression in autoimmunity.

## Results

### Magnetic CD4 sort effectively purifies splenic cells into CD4^lo^ (Th40 cells) and CD4^hi^ T cells

We have previously purified CD4^lo^ and CD4^hi^ T cells by flow cytometric sorting [3]. However, this method is costly and time consuming which lead us to develope a magnetic sort (detailed in the methods section) that yielded CD4^lo^ and CD4^hi^ T cells. We determined levels of T cell associated molecules in the CD4^lo^ population. CD4^lo^ T cells from both NOD and BALB/c mice expressed CD4 and CD3 (Figure S1A–D). However, portions of those proteins were contained intracellularly (Figure S1A–D). Additionally, CD4^lo^ cells from both strains of mice expressed CD28 at similar mean fluorescence intensities as CD4^hi^ T cells (Figure S1E). The CD4^lo^ cells were further categorized as T cells by staining and RT-PCR for TCR. CD4^lo^ cells from NOD and BALB/c mice exhibited expression of an array of both TCRβ and TCRα molecules (Figure S2A and B). Additionally, CD4^lo^ T cells express IFNγ when activated (data not shown).

To address the possibility of B cell contamination the B cell specific proteins CD19 and CD21 were stained on CD4^lo^, CD4^hi^, and depleted cells (MHCII^+^, CD8^+^, and CD25^+^ cells; removed by sorting). The CD4^lo^ and CD4^hi^ T cell populations exhibited very low B cell contamination (Figure S3A). As expected, the majority of the depleted cells (MHCII^+^CD8^+^CD25^+^) expressed CD19 and CD21 (Figure S3A). We further determined levels of CD11b and CD11c which are mainly associated with monocytes/macrophages and dendritic cells, respectively, but have also been shown to be expressed by subsets of T cells [36]–[38]. A small portion of CD4^lo^ T cells from both NOD and BALB/c expressed CD11b (Figure S3B). In addition, when cultured in tissue culture flasks the CD4^lo^ T cells take on a morphology like that of T cell clones and the size of CD4^lo^ T cells, when activated, never increases like that of activated antigen presenting cells (data not shown).

### NOD CD4^lo^ T cells express high levels of CD40

**Figure 1:**
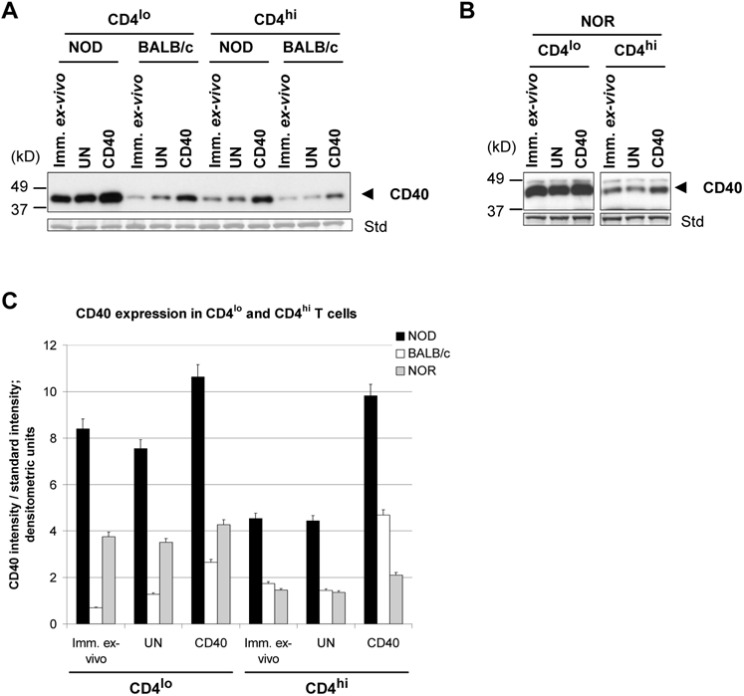
NOD Th40 cells express high levels of CD40 compared to BALB/c. Immediately *ex-vivo*, magnetically sorted Th40 cells and CD4^hi^ T cells from age matched 8–12 week-old female NOD (euglycemic), BALB/c or NOR were either immediately lysed (Imm. *ex-vivo*) or cultured overnight in the absence (UN) or presence of CD40 engagement (CD40) then lysed. Whole cell extracts, 5 µg, were analyzed by western blot for CD40. As an internal standard for loading (std) the membranes were stripped and stained with coomassie blue and a representative band is shown. (A) Western blot for CD40 in Th40 cells and CD4^hi^ T cells from NOD and BALB/c. (B) Western blot for CD40 in Th40 cells and CD4^hi^ T cells from NOR. (C) Graph representing the data in A and B. Data in C are represented as mean±SEM from at least 3 separate experiments.

**Figure 2:**
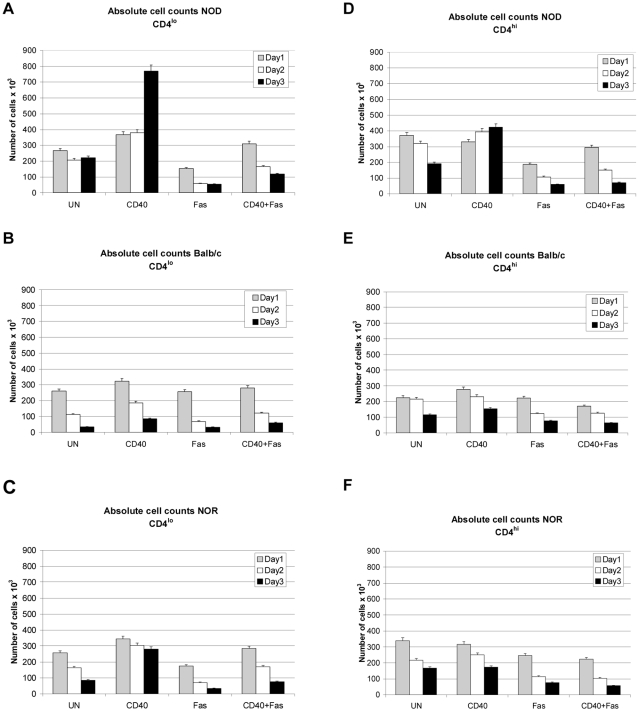
CD40 engagement of NOD Th40 cells induces increased survival. Sorted Th40 cells and CD4^hi^ T cells from age matched 8–12 week-old female NOD (euglycemic), NOR or BALB/c were untreated (UN), CD40-crosslinked (CD40), Fas crosslinked (Fas) or (CD40+Fas)-crosslinked ((CD40+Fas)) for 1, 2 and 3 days. Absolute numbers of live cells were counted by trypan blue exclusion. (A) NOD CD4^lo^. (B) BALB/c CD4^lo^. (C) NOR CD4^lo^. (D) NOD CD4^hi^. (E) BALB/c CD4^hi^. (F) NOR CD4^hi^. Data are represented as mean±SEM from 3 separate experiments.

We have shown that CD4^lo^CD40^+^ T cells are expanded in autoimmune prone mice [4], [15], [16] as well as in human T1D patients [17]. Therefore we determined whether there is also a difference on a per-cell basis in CD40 expression in those cells comparing autoimmune prone NOD to non-autoimmune BALB/c and NOR mice. NOR mice are diabetes resistant but contain greater than 90% of the NOD genome, including the unique IA^g7^ MHCII [39]. Cells from NOD, NOR, and BALB/c mice were sorted into CD4^lo^ and CD4^hi^ T cells. In immediately *ex-vivo* CD4^lo^ cells from NOD mice CD40 expression was 12-fold higher than that in the same subset from BALB/c mice (Figure 1A and C) and 2-fold higher than that of NOR (Figure 1A–C; note that the standard in the NOR experiment (B) is more intense than the standard in the NOD and BALB/c experiment (A)). CD4^hi^ T cells from all three mouse strains expressed CD40 at a lower level than CD4^lo^ T cells but, by direct comparison, CD4^hi^ T cells from NOD mice had higher CD40 levels than NOR and BALB/c CD4^hi^ T cells (about 2.5-fold; Figure 1A–C). When CD40 was engaged for 20 hrs, both populations from NOD, BALB/c, and NOR mice induced an increase in CD40 expression demonstrating a positive feedback loop.

### CD40 signals aid in survival of NOD Th40 cells *in vitro*

Th40 cells are expanded in autoimmune mouse strains [4], [15] and that expansion is prevented in NOD mice by blocking CD40–CD154 interactions [4]. Therefore we determined whether CD40 signals lead directly to increased survival of Th40 cells *in vitro* and whether there is a difference between autoimmune and non-autoimmune derivation of the Th40 cells.

Sorted Th40 cells (CD4^lo^) and CD4^hi^ T cells, from spleens of NOD, NOR and BALB/c mice, were cultured in the absence or presence of CD40-, Fas- or (CD40+Fas)-engagement then absolute numbers of live cells were counted after 1, 2, and 3 days of culture. It was evident that CD40 engaged NOD Th40 cells, compared to untreated, survived more readily over 3 days (Figure 2A). In fact, the number of cells doubled. In contrast there was no clear benefit of CD40 engagement in the same population from BALB/c mice (Figure 2B). Th40 cells from NOR survived better, compared to untreated, when CD40 engaged (Figure 2C). However, the survival induced by CD40 declined with time (Figure 2C). When Fas was engaged on NOD Th40 cells, cell death was induced (Figure 2A). Interestingly, if CD40 was engaged simultaneously with Fas, the Fas-induced death was thwarted, most prominently on day 1 (Figure 2A; p<0.001, t-test) persisting even 3 days after stimulation (Figure 2A; p<0.05, t-test). Similarly, NOR Th40 cells were rescued from Fas induced death by CD40 engagement but the absolute number of cells from NOD mice that survived was 1.5 times greater than those from NOR mice after 3 days (Figure 2A and C). The same type of rescue was not detected in BALB/c Th40 cells (Figure 2B). In NOD CD4^hi^ T cells, CD40 engagement led to somewhat increased survival (Figure 2D). However, this survival was not accompanied by a drastic increase in the number of cells. While there was a CD40-induced rescue from Fas death in the NOD CD4^hi^ population on day 1 (Fig. 2D; p<0.05, t-test), that rescue was not sustained on day 3 (p>0.6, t-test). BALB/c and NOR CD4^hi^ T cells did not preferentially survive in response to CD40 signals (Fig. 2E and F). These data demonstrate a crucial CD40 signaling difference, relative to cell survival, in the potentially autoaggressive Th40 cell population between autoimmune and non- autoimmune conditions.

### Potentially autoaggressive Th40 cells from NOD proliferate in response to CD40 engagement

**Figure 3:**
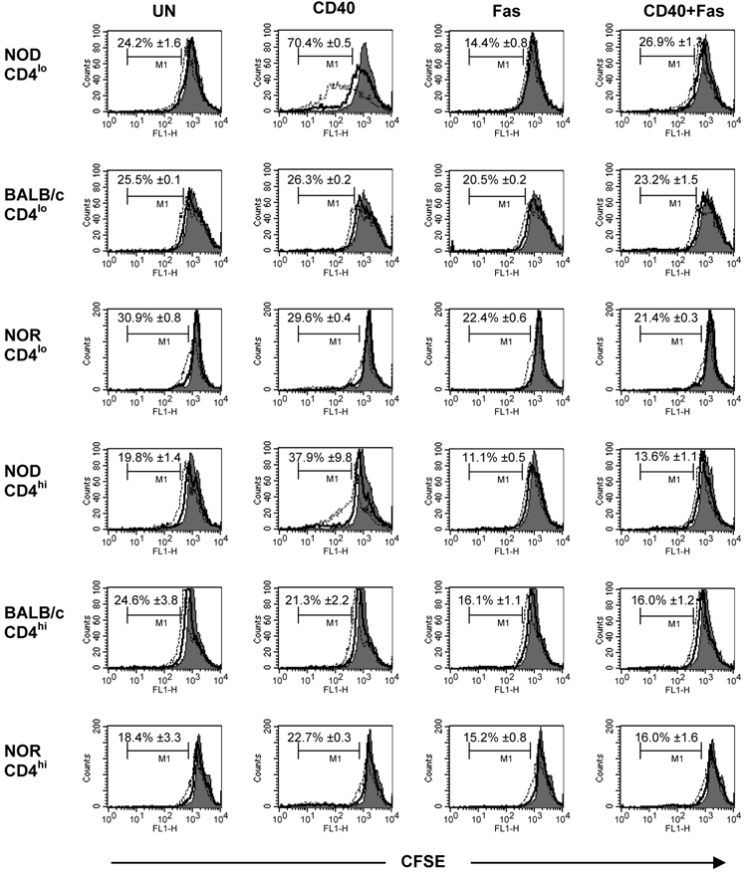
CD40 engagement of NOD Th40 cells induces proliferation. Sorted Th40 cells and CD4^hi^ T cells from age matched 8–12 week-old female NOD (euglycemic), NOR or BALB/c were labelled with CFSE and were then untreated (UN), CD40-crosslinked (CD40), Fas-crosslinked (Fas) or (CD40+Fas)-crosslinked ((CD40+Fas)) for 1, 2 and 3 days. T cell proliferation was assessed by CFSE dilution on day 1 (filled histogram), day2 (solid line) and day 3 (dashed line) in FACS analysis on ungated cells. Percentages represent amount of cells that proliferated on day 3 and are represented as mean±SEM from 3 separate experiments.

Because the NOD Th40 cells readily survived and nearly doubled in numbers in response to CD40 signals *in vitro*, we determined the effect of CD40 engagement on proliferation. Untreated NOD Th40 cells underwent minimal proliferation over 3 days in culture (Fig. 3, UN). However, when cells were CD40 engaged for 3 days significant proliferation was seen (Fig. 3, CD40). When NOD Th40 cells were Fas engaged, the minimal proliferation seen in untreated cells was reduced (Fig. 3, compare UN and Fas). When CD40 was engaged simultaneously with Fas, NOD Th40 cells proliferated to the same extent as untreated cells but not to the extent seen with CD40 treatment alone (Fig. 3). This demonstrates that although CD40 signals thwarted Fas-induced death, as seen in figure 2, Fas signals ablated the proliferation seen in CD40 stimulated NOD Th40 cells. Unlike T cells from NOD mice, NOR and BALB/c Th40 cells did not proliferate in response to CD40 (Fig. 3, compare UN and CD40). Additionally, CD4^hi^ T cells from NOD, but not NOR and BALB/c mice, demonstrated some proliferation in response to CD40 engagement (Fig. 3, CD40). However, this did not lead to a significant expansion of these cells as shown in figure 2.

These data demonstrate that CD40 engagement alone can drive not only survival but also expansion of the potentially autoaggressive Th40 cells in autoimmune NOD, but not in non-autoimmune NOR and BALB/c mice. These intrinsic differences relative to CD40, and now Fas, signaling suggest a mechanism to promote autoimmunity.

### CD40 and TRAF2 raft microdomain distribution is different between autoimmune and non-autoimmune Th40 cells

**Figure 4:**
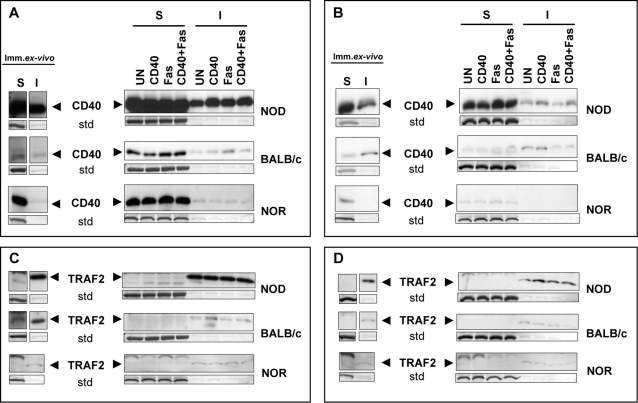
Immediately *ex-vivo* NOD Th40 cells have CD40 and TRAF2 associated with rafts. Sorted Th40 cells and CD4^hi^ T cells from age matched 8–12 week-old female NOD (euglycemic), BALB/c or NOR were immediately lysed (Imm. *ex-vivo*) or untreated (UN), CD40-crosslinked (CD40), Fas-crosslinked (Fas) or (CD40+Fas)-crosslinked ((CD40+Fas)) for 5 hours then soluble (S) and insoluble (I) fractions were prepared. CD40 levels in Th40 cells and CD4^hi^ (A and B respectively) and TRAF2 levels in Th40 cells and CD4^hi^ (C and D respectively) were analyzed in western blots. CD40 was analyzed first then the membranes were stripped and TRAF2 was analyzed. As internal loading standard (std) membranes were stripped and stained with coomassie blue and a representative band of the same size from each membrane is shown. Data are representative of 3 separate experiments.

When CD40 is engaged on both B and T cells it becomes associated with lipid raft microdomains to interact with adaptor molecules, TRAFs, for downstream signaling [6], [11]. Therefore we determined whether CD40 became associated with the detergent-insoluble raft fraction in the Th40 cells and CD4^hi^ T cells from NOD, NOR, and BALB/c mice when CD40 was engaged. Strikingly, a large amount of CD40 was associated with the raft fraction in immediately *ex-vivo* NOD Th40 cells compared to only a small amount in Th40 cells from NOR and BALB/c (insoluble fraction; Fig. 4A). When CD40 was engaged, this association increased slightly in NOD Th40 cells (Fig. 4A). In immediately *ex-vivo* CD4^hi^ T cells from NOD and BALB/c mice, association of some CD40 with the raft fraction was observed while in the same subset from NOR mice CD40 was not detected in this fraction (Fig. 4B). CD40 engagement of CD4^hi^ T cells from both NOD and BALB/c mice sustained the association of CD40 with the raft fraction (Fig. 4B). These data demonstrate that, given the immediately *ex-vivo* data, NOD Th40 cells likely are CD40 engaged *in vivo*. Therefore these cells may be poised for survival and proliferation should they encounter death promoting signals such as Fas stimulation.

TRAFs, including TRAF2, function as CD40 signal adaptor molecules in both B and T cells and associate with lipid raft microdomains upon CD40 stimulation [6], [11], [40]. Therefore we determined if TRAF2 was present in the raft fraction, setting the stage for CD40 signaling. TRAF2 was strongly associated with the raft fraction in NOD Th40 cells and remained associated during culture regardless of stimulation (Fig. 4C). However, in NOR and BALB/c Th40 cells, only a small amount of TRAF2 was associated with the raft fraction immediately *ex-vivo* (Fig. 4C). In CD4^hi^ T cells from all three mouse strains smaller amounts of TRAF2 associated with the raft fraction compared to that seen in the Th40 cells (Fig. 4D compared to C). NOD CD4^hi^ T cells had higher amounts compared with NOR and BALB/c CD4^hi^ T cells (Fig. 4D). This demonstrates that NOD Th40 cells have the signaling machinery necessary to transmit CD40 signals associated with the raft microdomain immediately *ex-vivo*. This could be due to the high availability of CD154 in autoimmunity [1], [41]–[44] that can constantly engage CD40 on these T cells.

### CD40 engagement induces increased, Nf-κB dependent, expression of Bcl-X_L_ in Th40 cells but only NOD Th40 cells induce cFLIP_p43_

Increased expression of anti-apoptotic factors such as Bcl-X_L_, which affects the intrinsic pathway, have been implicated in decreased apoptosis. Autoimmune diseases characteristically maintain T cells that are resistant to cell death [45]–[47]. Cleaved cFLIP (cFLIP_p43_) is protective in the extrinsic pathway and interacts with TRAF2 to induce Nf-κB activation [34]. Given that NOD Th40 cells readily survive in response to CD40 engagement we examined whether there were differences in Bcl-X_L_ and cFLIP expression between Th40 cells from NOD, NOR and BALB/c mice.

**Figure 5:**
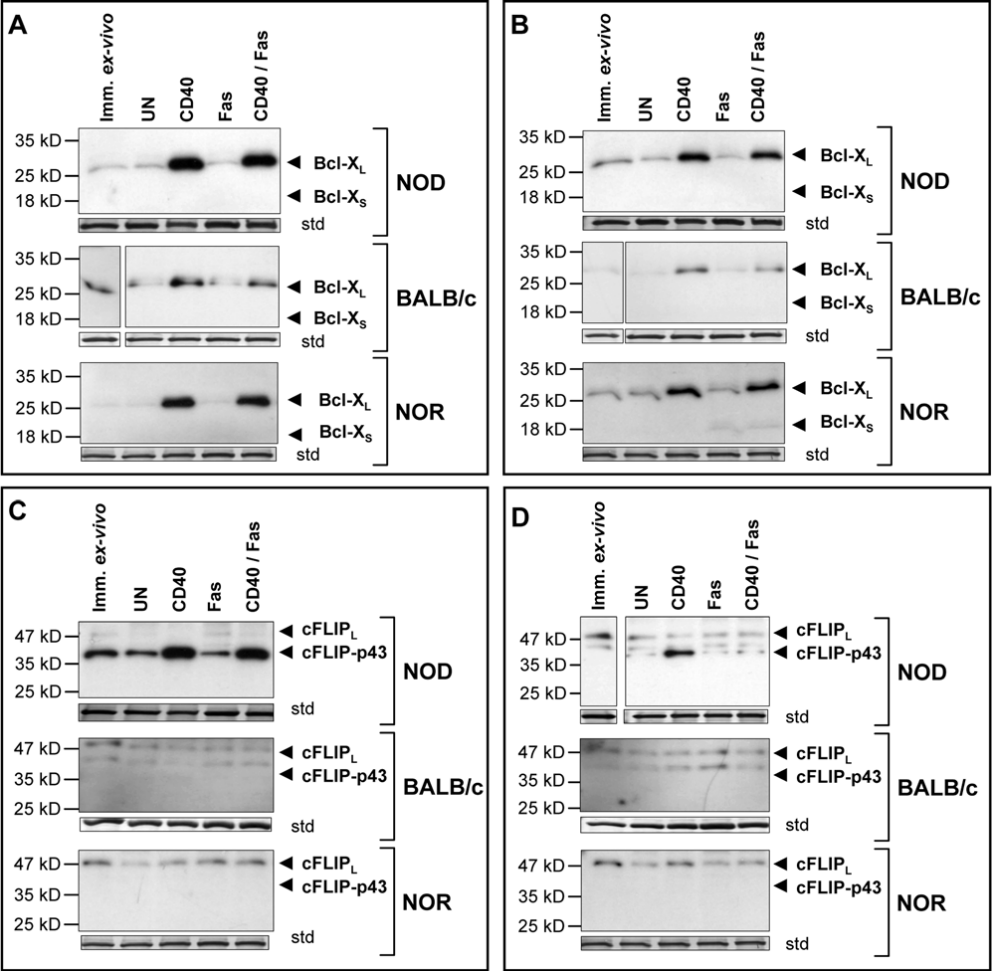
CD40 engagement induces high Bcl-X_L_ and cFLIP_p43_ expression in NOD Th40 cells. Sorted Th40 cells and CD4^hi^ T cells from age matched 8–12 week-old female NOD (euglycemic), BALB/c or NOR were untreated (UN), CD40-crosslinked (CD40), Fas-crosslinked (Fas) or (CD40+Fas)-crosslinked (CD40+Fas) for 20 hours. Whole cell lysates from CD4^lo^ T cells (A and C) or CD4^hi^ T cells (B and D) were analyzed in western blot for Bcl-X or cFLIP. As internal loading standard (std), membranes were stripped and stained with coomassie blue and a representative band of the same size from each membrane is shown. Data are representative of 3 separate experiments.

A basal level of Bcl-X_L_ was apparent in both NOD and BALB/c, but not in NOR, Th40 cells (Fig. 5A and Figure S4). CD40 engagement induced high Bcl-X_L_ protein expression in NOD as well as NOR Th40 cells. In BALB/c Th40 cells more modest levels of Bcl-X_L_ were induced (Fig. 5A and Figure S4). CD4^hi^ T cells from NOD and NOR mice induced fairly high expression of Bcl-X_L_ in response to CD40 engagement although not as high as those of the respective Th40 cell subsets (Fig. 5A and B and Figure S4). Meanwhile, BALB/c CD4^hi^ T cells induced only low levels of Bcl-X_L_ (Fig. 5B and Figure S4). Interestingly, the CD40-induced Bcl-X_L_ expression was sustained in both cell subsets from all three mouse strains even when Fas was engaged simultaneously (Fig. 5A and B and Figure S4). Additionally, NOR CD4^hi^ T cells induced low levels of pro-apoptotic Bcl-X_S_ in response to Fas engagement (Figure 5B).

When examining cFLIP levels, NOD Th40 cells exhibited a basal expression of cFLIP_p43_, the activated, protective form of cFLIP (Figure 5C). When CD40 engaged, NOD Th40 cells induced higher expression of cFLIP_p43_ and this higher expression was sustained with simultaneous Fas engagement (Figure 5C). In NOR and BALB/c Th40 cells, however, only cFLIP_L_ was expressed and none of the treatments induced cFLIP_p43_ (Figure 5C). In CD4^hi^ T cells from NOD, NOR and BALB/c mice, a low basal level of cFLIP_L_ was observed but only in NOD CD4^hi^ T cells was cFLIP_p43_ expression induced in response to CD40 engagement (Figure 5D). The induced cFLIP_p43_ expression was not sustained when Fas was simultaneously engaged.

The data demonstrate that CD40 successfully signals to both Th40 and CD4^hi^ T cell subsets in NOD, NOR, and BALB/c mice to induce Bcl-X_L_ expression. That induced expression appears independent of Fas signals. In the highest expressors, i.e. Th40 cells from NOD and NOR, Bcl-X_L_ expression appears to protect against Fas induced cell death. However, over time NOD Th40 cells appear to survive more readily (Figure 2) perhaps due to the high levels of induced cFLIPp_43_. In one of the moderate expressors of Bcl-X_L_, i.e. CD4^hi^ T cells from NOD, CD40 engagement initially induces rescue from Fas death. However, with time and with CD40 induced cFLIP_p43_ expression being all but thwarted when Fas was engaged simultaneously, the benefit is no longer there. NOR CD4^hi^ T cells are also moderate Bcl-X_L_ expressors but are not rescued from Fas death by CD40 engagement. This is perhaps due to that Bcl-X_S_, a pro-apoptotic protein, is induced by Fas engagement in the NOR CD4^hi^ T cells. Relative to proliferation it appears that only those cells capable of inducing cFLIP_p43_ expression, i.e. NOD Th40 cells and to a lesser extent NOD CD4^hi^ T cells, are able to proliferate.

**Figure 6:**
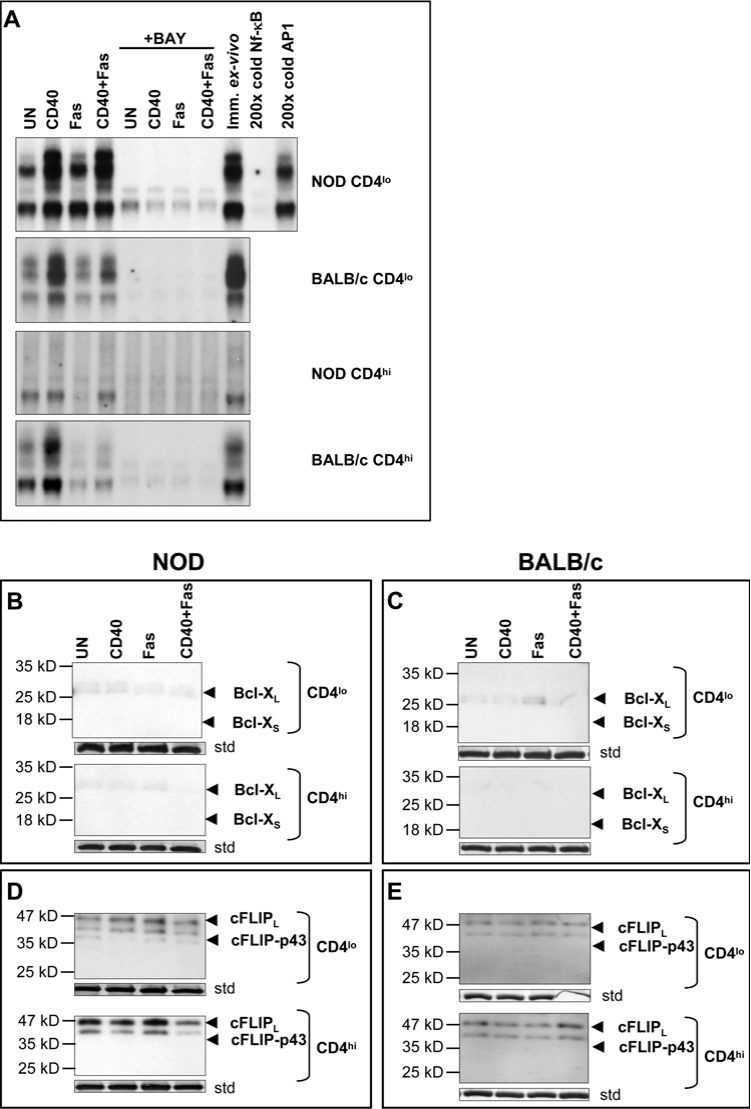
CD40 induces Nf-kB activation in Th40 cells and Nf-kB inhibition ablates CD40-induced Bcl-X_L_ and cFLIP_p43_. Sorted Th40 cells and CD4^hi^ T cells from age matched 8–12 week-old female NOD (euglycemic) or BALB/c were untreated (UN), CD40-crosslinked (CD40), Fas crosslinked (Fas) or (CD40+Fas)-crosslinked ((CD40+Fas)) for 20 hours in the absence/presence of the Nf-kB inhibitor BAY 11-7082 (BAY). (A) Nuclear extracts were analyzed in EMSA for Nf-kB DNA-binding activity using a consensus Nf-kB oligonucleotide. As controls cold Nf-kB or AP1 oligonucleotides were used in competiotion reactions. Free oligonucleotide is not shown. Data represent 3 separate experiments. (B) Whole cell lysates from BAY 11-7082 treated NOD (B and D) or BALB/c (C and E) were analyzed in western blot for Bcl-X or cFLIP. As internal loading standard (std), membranes were stripped and stained with coomassie blue and a representative band of the same size from each membrane is shown. Data represent 3 separate experiments.

Bcl-X_L_ and cFLIP expression are dependent on Nf-κB activation [48], [49]. Since we have shown that CD40 engagement activates Nf-κB in a T cell clone [16] we determined whether CD40 could induce Nf-κB activation in primary T cells as well. CD40 engagement induced Nf-κB DNA-binding activity in primary Th40 cells from both NOD and BALB/c mice although the induction was greater in NOD Th40 cells (Figure 6A). The CD40-induced Nf-κB DNA-binding activity in NOD Th40 cells was sustained when Fas was simultaneously engaged (Figure 6A). Interestingly, in BALB/c Th40 cells, Fas engagement blocked most of the CD40-induced Nf-κB DNA-binding activity. CD4^hi^ T cells from both BALB/c and NOD mice demonstrated little or no Nf-κB DNA-binding activity in response to CD40 engagement (Figure 6A). When an Nf-κB inhibitor, BAY 11-7082 [50], [51], was included during the different treatments all Nf-κB DNA-binding activity was abolished regardless of treatment and cell population (Figure 6A, “+BAY”).

We analyzed the levels of Bcl-X_L_ and cFLIP in the presence of the Nf-kB inhibitor. In the presence of BAY 11-7082 neither cell population from NOD or BALB/c mice expressed Bcl-X_L_ (Figure 6B and C). Likewise, cFLIP_p43_ expression was completely abolished in the presence of BAY 11-7082 while the long form of cFLIP, cFLIP_L_, was more readily expressed (Figure 6D and E). This indicates that Nf-κB DNA-binding activity is necessary for the expression of survival proteins in response to CD40 engagement. Additionally, given the strong expression of TRAF2 in the raft microdomain of NOD Th40 cells (Figure 4), the possibility for interaction of induced cFLIP_p43_ with TRAF2 and further activation of Nf-κB, as has been demonstrated [34], arises.

## Discussion

Recently, reports have focused on the importance of T cell homeostasis [4], [26], [52]–[56] in protection against autoimmunity. Although the focus has been mainly on regulatory T cells, we have demonstrated the importance of the ratio of potentially autoaggressive Th40 cells to T_reg_ [4]. In fact, we determined that an intricate balance between T_regs_ and potentially pathogenic Th40 cells must be maintained to prevent autoimmunity and this process is tightly associated with CD40 signaling [57]. Autoimmune prone animals, and incidentally human T1D patients [17], have a substantial abundance of peripheral Th40 cells which are highly pathogenic in autoimmune conditions [4], [15], [16]. However, the disrupted homeostasis in autoimmune prone NOD mice can be prevented by blocking CD40–CD154 interaction [4], [5]. In the present study we further define differences in this dyad by showing that not only are there differences in CD40 expression levels between autoimmune and non-autoimmune conditions but the microdomain distribution of CD40, as well as of the signaling adaptor molecule TRAF2, is different in the potentially autoaggressive Th40 cell subset. The CD40 signaling outcome is consequently different between autoimmune and non-autoimmune conditions. Clearly, with only the need for CD40 engagement, the Th40 cells from autoimmune prone NOD mice are poised for survival and proliferation via activation of Nf-κB DNA-binding activity and expression of high levels of anti-apoptotic proteins Bcl-X_L_ and cFLIP_p43_.

A striking finding is that the CD40 engaged NOD and NOR Th40 cells could thwart Fas-induced death. However, only the NOD Th40 cells survived and proliferated. Therefore it is possible that CD40-induced Nf-κB activation could ultimately, via induction of Bcl-X_L_ expression and via decoy activity and further Nf-κB activation by cFLIP_p43_, keep the culprit autoaggressive Th40 cells resistant to death in autoimmunity and further allow expansion of this T cell subset. Interestingly, it was shown that NOR splenic T cells depleted of T_reg_ transferred diabetes to NOD.scid recipients [57], [58] and we have recently transferred diabetes to NOD.scid recipients using NOR Th40 cells [57]. Therefore it appears that NOD and NOR Th40 cells share the CD40 hyperexpression and pathogenic TCRs that predisposes them to become autoaggressive. However, the molecular environment in which they exist differs, as shown here, to favor CD40 signaling in NOD Th40 cells which poises them for survival and proliferation by constantly having CD40 and TRAF2 mobilized to the raft. Once the predisposed Th40 cells from NOR mice are transferred into the autoimmune environment, however, they are triggered to proliferate and become autoaggressive although at a much slower pace than the same subset from NOD [57].

While the CD40 receptor on Th40 cells from non-autoimmune BALB/c mice is funtional, as demonstrated by the induction of Nf-kB activation as well as Bcl-X_L_ expression, it was not capable of induction of resistance to Fas death or of inducing proliferation. This demonstrates clear mechanistic differences relative to CD40-signaling in autoimmune versus non-autoimmune conditions. Additionally, it indicates that the CD40-signaling pathway that leads to cFLIP_p43_ expression could be targeted to control the Th40 cells in autoimmunity.

Genetic linkage studies do not indicate CD40 as a candidate gene in autoimmunity. However, the present data demonstrate that conditions prevailing in autoimmunity lead to hyperexpression of CD40 on the potentially autoaggressive Th40 cell subset. This in turn leads to alterations in the signaling of CD40 and ultimately in life-and-death decisions in those cells. It is possible that a genetic deviation in a different gene leads to those conditions and that CD40 therefore by proxy is a culprit in autoimmune disease.

Intrinsic T_reg_, i.e. specifically described as CD4^+^CD25^+^foxP3^+^ T cells [26], [52]–[56], which prevent autoimmune disease, are expanded in NOD mice only after blocking CD40–CD154 interaction [4]. This together with the present data suggests that homeostasis, specifically some maintained balance between T_reg_ and potentially autoaggressive Th40 cells, can be CD40 regulated in autoimmune mice. Given the apparent hyperexpression of CD40 on Th40 cells and the increased availability of CD154 in autoimmune conditions [1], [41]–[44], homeostasis can readily be broken as has been demonstrated [4], [16]. The specific conditions leading to the observed differences are being studied and may hold clues to how to control the culprit T cells in autoimmunity and provide novel treatments and prevention of autoimmune disease.

## Materials and Methods

### Mice

NOD, NOR, and BALB/c mice from Jackson Laboratories, Bar Harbor, Maine, were maintained under pathogen-free conditions at the Webb-Waring Institute, University of Colorado HSC, IACUC-approved facility. The NOD colony consistently achieves >90% diabetes in females by the age of 18 weeks. All experiments were carried out under IACUC-approved protocol number 55802006(04)1E.

### Antibodies and reagents

Anti-CD40 (sc-975) and anti-Bcl- X_L/S_ (sc-634) were from Santa Cruz Biotechnology, Inc., Santa Cruz, California, and anti-cFLIP (F9800) from Sigma, St. Louis, MO. Biotinylated anti-Fas (Jo-2) was from BD Biosciences, San Diego, CA. Biotinylated anti-CD40 antibodies (1C10 and 4F11) were produced in-house. Biotinylated anti-Fas and anti-CD40 (1C10 and 4F11 always used together) antibodies were used at 5 µg/ml each followed by 1.0 µg/ml streptavidin to crosslink. NF-κB blocker, BAY 11-7082, was from BIOMOL Research Laboratories, Inc., Plymouth Meeting, PA, and was used at 10 µM. Biotinylated anti-CD25 (7D4) and anti-CD4 (GK1.5) were produced in-house. Anti-CD8-, anti-MHCII-, and streptavidin-conjugated magnetic microbeads were from Miltenyi Biotec Inc., Auburn, California.

### T cell purification and cell culture

Spleens from age matched female 8–12 week old NOD, NOR and BALB/c mice were homogenized in red blood cell lysis buffer (Sigma-Aldrich, St. Louis, Missouri) then pelleted. The cell pellet was resuspended in 1 ml (per original spleen) PBS containing 2% BSA and 2 mM EDTA. This buffer was used in all incubations and washes. Biotin-anti-CD25, 1 µg/µl, was added at 20 µl/spleen and incubated, rocking, for 15 minutes at room temperature. Streptavidin-, anti-CD8-, and anti-MHCII-microbeads were added at 20, 40, and 40 µl/spleen, respectively, and then incubated another 15 minutes. Cells were washed once with 10 ml buffer then sorted in “DepleteS” (slow-flow) mode using an autoMACS™ magnetic cell sorter (Miltenyi Biotec Inc., Auburn, California). Depleted cells (CD25^−^CD8^−^MHCII^−^) were labeled with biotin-anti-CD4, 1 µg/µl, at 40 µl/spleen for 15 minutes then washed once as above. Streptavidin-microbeads, 40 µl/spleen, were added, incubated for another 15 minutes then cells were washed once. Cells were sorted in “Possel” (fast-flow) in autoMACS™. Magnetically positive cells were considered CD4^hi^ and magnetically negative cells were considered Th40 cells (CD4^lo^CD40^+^). (While Th40 cells are magnetically CD4-negative they do express CD4, predominantly intracellularly, CD3 and TCR (Figure S1 and Figure S2).) Cells were cultured in DMEM containing 10% fetal calf serum and 50 µM β-mercaptoethanol. Cells were CD40- and/or Fas-crosslinked using 5 µg/ml each of biotinylated 1C10+4F11 and/or Fas, respectively, followed by 1.0 µg/ml of streptavidin to crosslink.

### T cell survival and proliferation assays

T cells were purified as described above then cultured in the absence/presence of CD40 crosslinking. After 1, 2, and 3 days live cells were counted by trypan blue exclusion to determine the survival rate. For proliferation assays the cells were labelled with CFSE, 5 µM, crosslinked as above and assayed after 1, 2, and 3 days on a FACSCalibur flowcytometer (BD Biosciences, San Diego, CA) for CFSE dilution.

### EMSA

Nuclear protein was prepared and assayed as described [16] on T cells that were purified and cultured as above. For the binding reaction 1.5 µg of nuclear protein was used with a radiolabeled NF-κB consensus oligonucleotide (Promega Corporation, Madison, WI).

### Western blot

Preparation of detergent-insoluble microdomains (rafts) from equal numbers of Th40 cells and CD4^hi^ T cells from NOD, NOR, and BALB/c was done as described [6]. Briefly, 1.9×10^6^ cells were lysed in 50 µl Brij-58 buffer and insoluble matter pelleted. The pellet was then solubilized in 50 µl octylglucopyranoside buffer. The resulting fractions were analyzed in western blots for CD40 and TRAF2 expression (10 µl/lane). Preparation of whole cell lysates from Th40 cells and CD4^hi^ cells was done by lysis in buffer (1% Triton X-100, 150 mM NaCl, 20 mM Tris, pH 7.5, 2 mM EDTA, 1 µg/ml each aprotinin and leupeptin, 0.4 mM PMSF, 0.4 mM sodium-ortho-vanadate, 0.5 mM DTT) for 10 minutes at room temperature then insoluble debris was pelleted. Bcl-X and cFLIP levels were analyzed by western blot, 10 µg protein/lane [3]. As internal loading standard, membranes were stripped and stained with Coomassie Blue R-250. Analysis of band intensities was done with Kodak 1D densitometry analysis software (Eastman Kodak Company, Rochester, New York).

## Supporting Information

Figure S1 CD4^lo^ cells express T cell associated proteins CD4, CD3 and CD28. NOD and BALB/c splenic cells were magnetically sorted into CD4^lo^ and CD4^hi^ populations as detailed in the methods section. (A) CD4^lo^ and CD4^hi^ cells were either stained immediately after sort (black lines) or cultured overnight then stained for CD4 (dashed lines). Grey-shaded histogram is isotype control. (Staining after overnight culture was done because the CD4-molecule on the CD4^hi^ cells was somewhat blocked for stain by the antibody used for sort (GK1.5). It is known that the antibody used for staining here (H129.19; CyChrome-conjugated from BD Bioscience) competes with the GK1.5 antibody.) Percentages on the left represent the amount of cells staining in the CD4-low range (M1) and on the right the amount of cells staining in the CD4-high range (M2) after overnight culture. Events were ungated. (B) Western blot for CD4 on whole cell extracts (10 ug/lane) from CD4^lo^ and CD4^hi^ cells was performed on cells immediately after sort using CD4 antibody (sc-1140) from Santa Cruz Biotechnology, Inc. As a loading control membranes were stripped and coomassie blue stained and a representative band is shown (std). (C) CD4^lo^ and CD4^hi^ cells were stained immediately after sort for CD3-ε (145.2C11; CyChrome-conjugated from BD Bioscience; black line). Grey-shaded histogram is isotype control. Percentages on the left represent the amount of cells staining in the CD3-low range (M1) and on the right the amount of cells staining in the CD3-high range (M2). Events were ungated. (D) Western blot for CD3, using CD3 antibody (sc-1127) from Santa Cruz Biotechnology, performed as above. (E) CD4^lo^ (black line) and CD4^hi^ (dashed line) cells were stained for CD28 (37.51; PE-conjugated from eBioscience) immediately after sort. Grey-shaded histogram is isotype control. Percentages represent the amount of cells staining in M1. Events were ungated.
(0.53 MB PPT)

Figure S2 CD4^lo^ cells express T cell receptor α and β. NOD and BALB/c splenic cells were magnetically sorted into CD4^lo^ and CD4^hi^ populations as detailed in the methods section. (A) CD4^lo^ and CD4^hi^ cells were stained immediately after sort for TCRβ (H57-597; CyChrome-conjugated from BD Bioscience; black line). Grey-shaded histogram is isotype control. Percentages on the left represent the amount of cells staining in the TCR-low range (M1) and on the right the amount of cells staining in the TCR-high range (M2). Events were ungated. (B) RT-PCR was performed on RNA from CD4^lo^ and CD4^hi^ cells from NOD and BALB/c using TCR Vα specific primers (Blish, C. A., B. J. Gallay, et al. (1999). J Immunol 162(6): 3131–40) or TCR Vβ specific primers (DiLorenzo, T. P., R. T. Graser, et al. (1998). Proc Natl Acad Sci U S A 95(21): 12538–43). Each lane had cDNA-starting-material equivalent to 750 cells.
(4.68 MB PPT)

Figure S3 CD4^lo^ T cells are not contaminated by B cells and a portion of the CD4^lo^ T cells express CD11b. NOD and BALB/c splenic cells were magnetically sorted into CD4^lo^ and CD4^hi^ populations as detailed in the methods section. The non-CD4 cells (MHCII^+^CD8^+^CD25^+^) initially depleted in the sort were kept for staining. (A) CD4^lo^, CD4^hi^ and MHCII^+^CD8^+^CD25^+^ cells were stained for CD21 and CD19 (eBio8D9 and MB19-1; PE- and PE-Cy5-conjugated, respectively, from eBioscience). Percentages represent cells staining in upper right quadrant. Events were ungated. (B) CD4^lo^ T cells from NOD and BALB/c were stained for CD11c and CD11b (N418 and M1/70; FITC- and PE-conjugated respectively.) Percentages represent cells staining in upper left quadrant. Events were ungated. Quadrants in A and B were set based on isotype controls.
(0.26 MB PPT)

Figure S4 Bcl-X_L_ expression in CD4^lo^ and CD4^hi^ T cells. Graph representing the data in figure 5A and B. Data are represented as mean±SEM from 3 separate experiments.
(0.07 MB PPT)
