# Supplementary material for: High Distribution of CD40 and TRAF2 in Th40 T Cell Rafts Leads to Preferential Survival of this Auto-Aggressive Population in Autoimmunity
Source: PLoS One. 2008 Apr 30;3(4):e2076. doi: 10.1371/journal.pone.0002076 (PMC2324204; doi:10.1371/journal.pone.0002076)
Supplement: Figure S1 — CD4lo cells express T cell associated proteins CD4, CD3 and CD28. NOD and BALB/c splenic cells were magnetically sorted into CD4lo and CD4hi populations as detailed in the methods section. (A) CD4lo and CD4hi cells were either stained immediately after sort (black lines) or cultured overnight then stained for CD4 (dashed lines). Grey-shaded histogram is isotype control. (Staining after overnight culture was done because the CD4-molecule on the CD4hi cells was somewhat blocked for stain by the antibody used for sort (GK1.5). It is known that the antibody used for staining here (H129.19; CyChrome-conjugated from BD Bioscience) competes with the GK1.5 antibody.) Percentages on the left represent the amount of cells staining in the CD4-low range (M1) and on the right the amount of cells staining in the CD4-high range (M2) after overnight culture. Events were ungated. (B) Western blot for CD4 on whole cell extracts (10 ug/lane) from CD4lo and CD4hi cells was performed on cells immediately after sort using CD4 antibody (sc-1140) from Santa Cruz Biotechnology, Inc. As a loading control membranes were stripped and coomassie blue stained and a representative band is shown (std). (C) CD4lo and CD4hi cells were stained immediately after sort for CD3-ε (145.2C11; CyChrome-conjugated from BD Bioscience; black line). Grey-shaded histogram is isotype control. Percentages on the left represent the amount of cells staining in the CD3-low range (M1) and on the right the amount of cells staining in the CD3-high range (M2). Events were ungated. (D) Western blot for CD3, using CD3 antibody (sc-1127) from Santa Cruz Biotechnology, performed as above. (E) CD4lo (black line) and CD4hi (dashed line) cells were stained for CD28 (37.51; PE-conjugated from eBioscience) immediately after sort. Grey-shaded histogram is isotype control. Percentages represent the amount of cells staining in M1. Events were ungated. (0.53 MB PPT) [file pone.0002076.s001.ppt]

## Slide 1
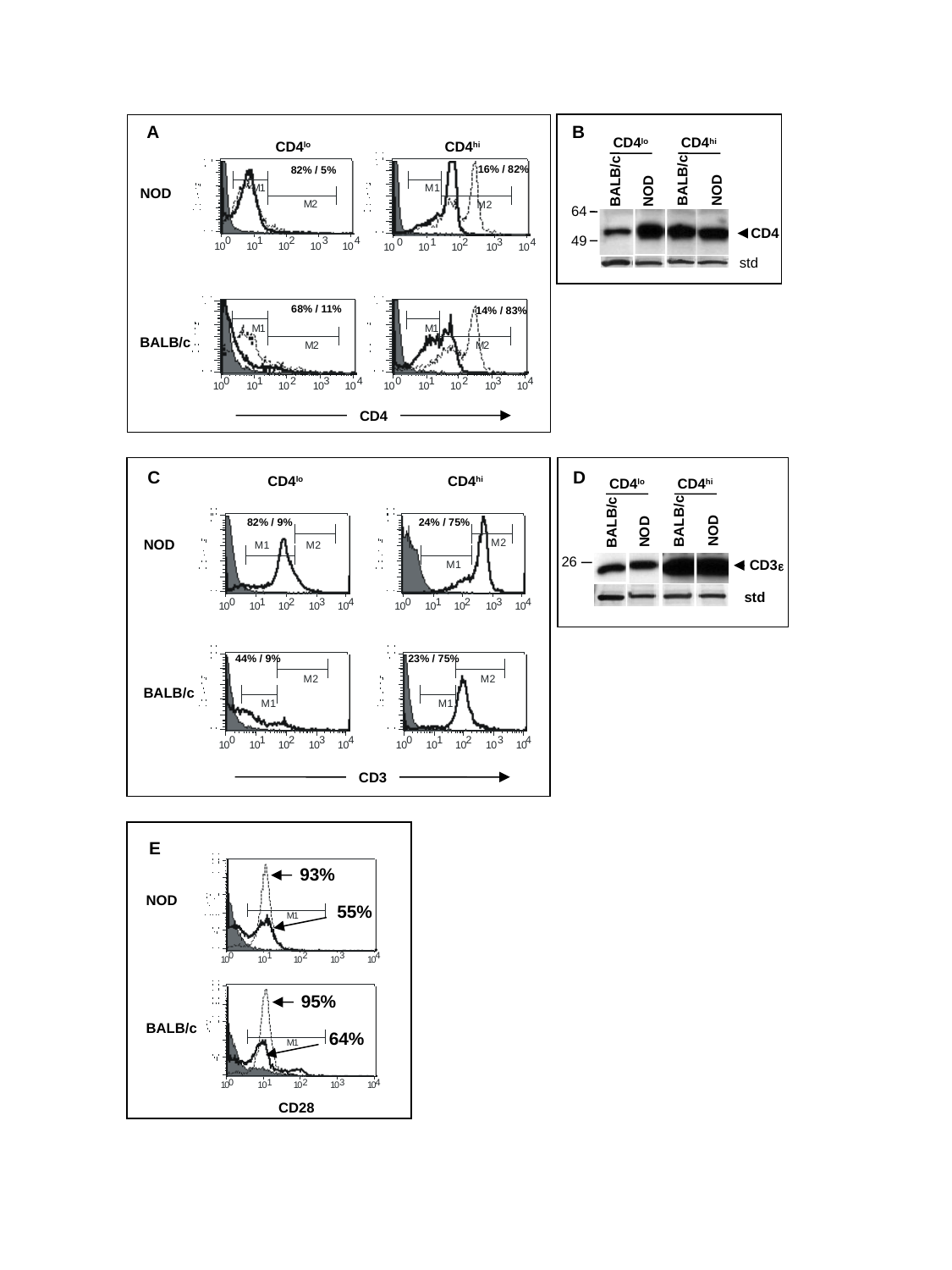

A
B
CD4lo
CD4hi
BALB/c
BALB/c
NOD
NOD
64
CD4
49
std
CD4lo
CD4hi
16% / 82%
82% / 5%
NOD
68% / 11%
14% / 83%
BALB/c
CD4
D
CD4lo
CD4hi
BALB/c
BALB/c
NOD
NOD
26
CD3ε
std
C
CD4lo
CD4hi
82% / 9%
24% / 75%
NOD
44% / 9%
23% / 75%
BALB/c
CD3
E
93%
NOD
55%
95%
BALB/c
64%
CD28
