# Supplementary material for: High Distribution of CD40 and TRAF2 in Th40 T Cell Rafts Leads to Preferential Survival of this Auto-Aggressive Population in Autoimmunity
Source: PLoS One. 2008 Apr 30;3(4):e2076. doi: 10.1371/journal.pone.0002076 (PMC2324204; doi:10.1371/journal.pone.0002076)
Supplement: Figure S2 — CD4lo cells express T cell receptor α and β. NOD and BALB/c splenic cells were magnetically sorted into CD4lo and CD4hi populations as detailed in the methods section. (A) CD4lo and CD4hi cells were stained immediately after sort for TCRβ (H57-597; CyChrome-conjugated from BD Bioscience; black line). Grey-shaded histogram is isotype control. Percentages on the left represent the amount of cells staining in the TCR-low range (M1) and on the right the amount of cells staining in the TCR-high range (M2). Events were ungated. (B) RT-PCR was performed on RNA from CD4lo and CD4hi cells from NOD and BALB/c using TCR Vα specific primers (Blish, C. A., B. J. Gallay, et al. (1999). J Immunol 162(6): 3131–40) or TCR Vβ specific primers (DiLorenzo, T. P., R. T. Graser, et al. (1998). Proc Natl Acad Sci U S A 95(21): 12538–43). Each lane had cDNA-starting-material equivalent to 750 cells. (4.68 MB PPT) [file pone.0002076.s002.ppt]

## Slide 1
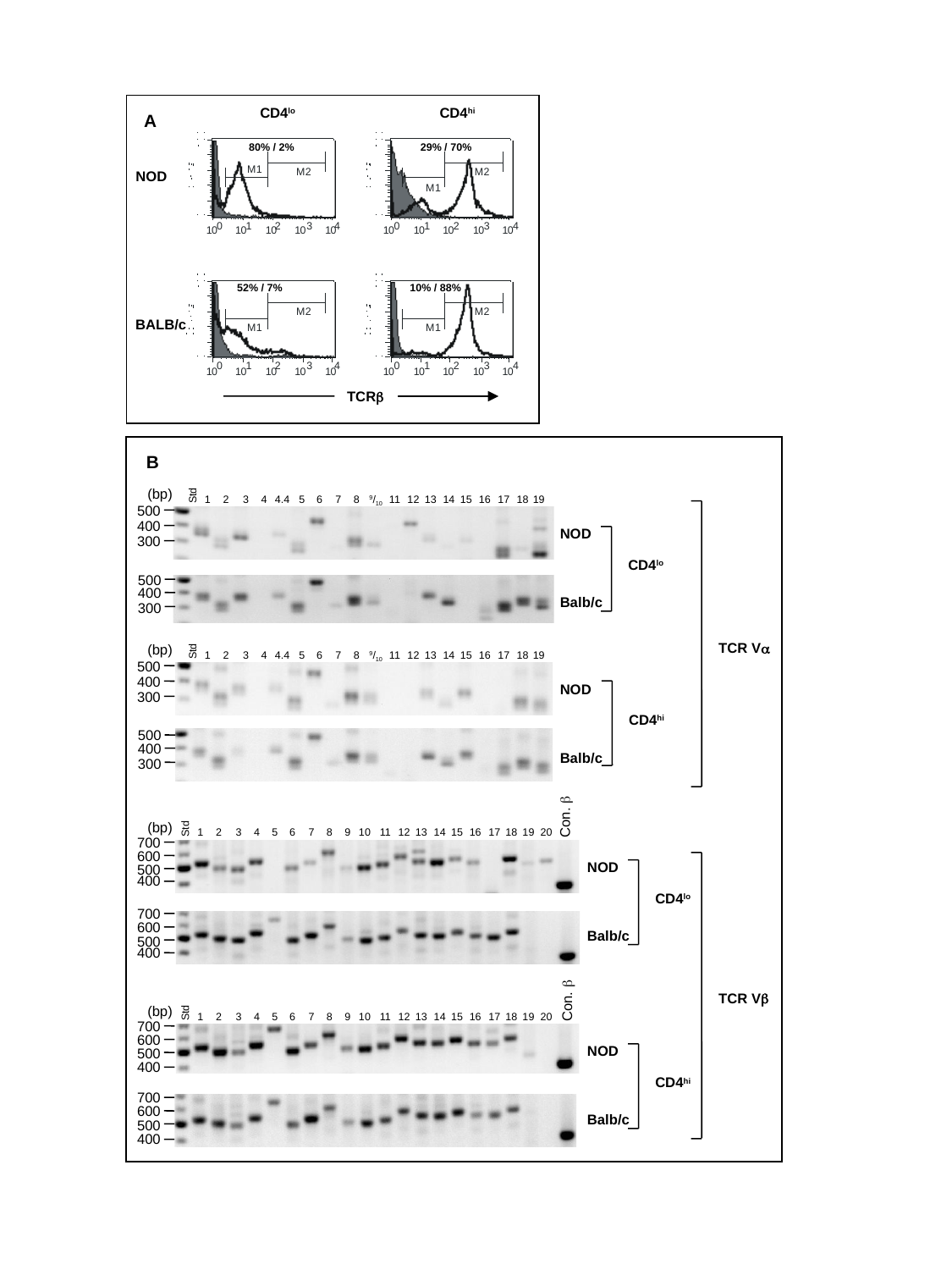

CD4lo
CD4hi
A
80% / 2%
29% / 70%
NOD
52% / 7%
10% / 88%
BALB/c
TCRβ
B
(bp)
Std
1
2
3
4
4.4
5
6
7
8
9/10
11
12
13
14
15
16
17
18
19
500
400
NOD
300
CD4lo
500
400
Balb/c
300
TCR Vα
(bp)
Std
1
2
3
4
4.4
5
6
7
8
9/10
11
12
13
14
15
16
17
18
19
500
400
NOD
300
CD4hi
500
400
Balb/c
300
Con. β
(bp)
Std
1
2
3
4
5
6
7
8
9
10
11
12
13
14
15
16
17
18
19
20
700
600
NOD
500
400
CD4lo
700
600
Balb/c
500
400
TCR Vβ
Con. β
(bp)
Std
1
2
3
4
5
6
7
8
9
10
11
12
13
14
15
16
17
18
19
20
700
600
NOD
500
400
CD4hi
700
600
Balb/c
500
400
