# Supplementary figures and images for: High Distribution of CD40 and TRAF2 in Th40 T Cell Rafts Leads to Preferential Survival of this Auto-Aggressive Population in Autoimmunity
Source: PLoS One. 2008 Apr 30;3(4):e2076. doi: 10.1371/journal.pone.0002076 (PMC2324204; doi:10.1371/journal.pone.0002076)

## Slide 1
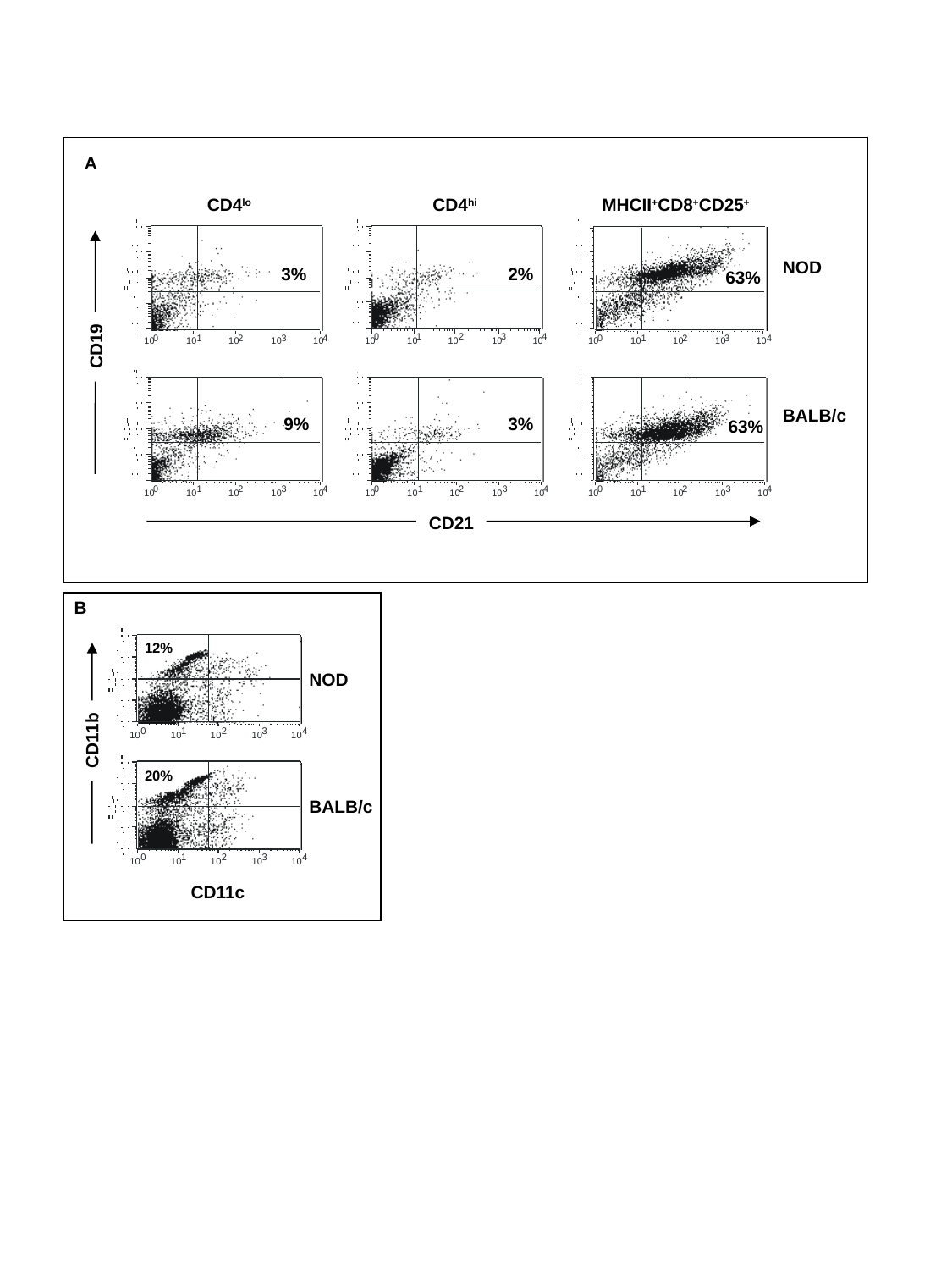

A
 CD4lo
 CD4hi
MHCII+CD8+CD25+
NOD
3%
2%
63%
CD19
BALB/c
9%
3%
63%
CD21
B
12%
NOD
CD11b
20%
BALB/c
CD11c

Supplement: Figure S3 — CD4lo T cells are not contaminated by B cells and a portion of the CD4lo T cells express CD11b. NOD and BALB/c splenic cells were magnetically sorted into CD4lo and CD4hi populations as detailed in the methods section. The non-CD4 cells (MHCII+CD8+CD25+) initially depleted in the sort were kept for staining. (A) CD4lo, CD4hi and MHCII+CD8+CD25+ cells were stained for CD21 and CD19 (eBio8D9 and MB19-1; PE- and PE-Cy5-conjugated, respectively, from eBioscience). Percentages represent cells staining in upper right quadrant. Events were ungated. (B) CD4lo T cells from NOD and BALB/c were stained for CD11c and CD11b (N418 and M1/70; FITC- and PE-conjugated respectively.) Percentages represent cells staining in upper left quadrant. Events were ungated. Quadrants in A and B were set based on isotype controls. (0.26 MB PPT) [file pone.0002076.s003.ppt]

## Slide 1
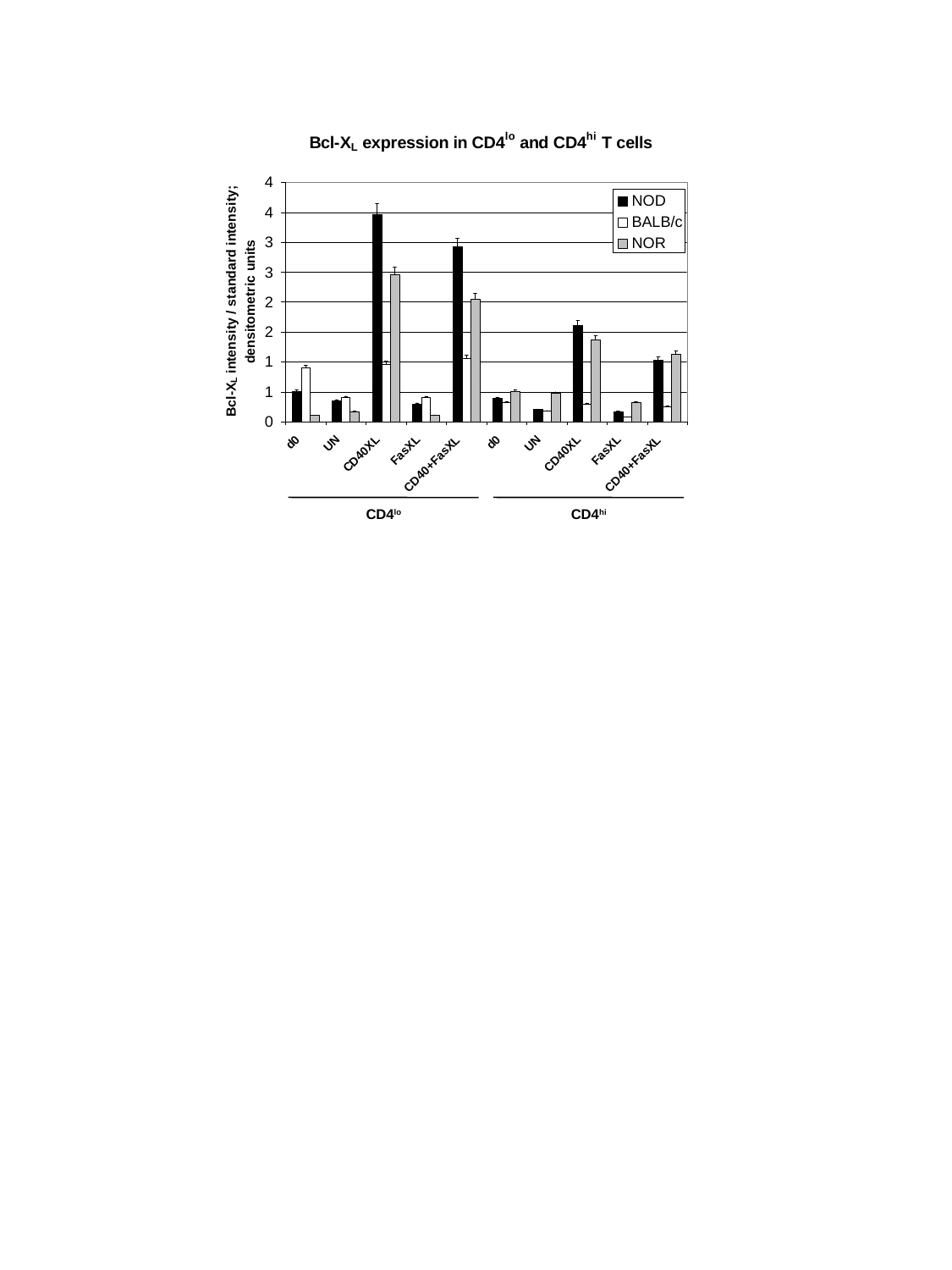

CD4lo
CD4hi

Supplement: Figure S4 — Bcl-XL expression in CD4lo and CD4hi T cells. Graph representing the data in figure 5A and B. Data are represented as mean±SEM from 3 separate experiments. (0.07 MB PPT) [file pone.0002076.s004.ppt]
